# Model systems of human immunodef iciency virus (HIV-1) for in vitro eff icacy assessment of candidate vaccines and drugs against HIV-1

**DOI:** 10.18699/VJGB-22-26

**Published:** 2022-03

**Authors:** N.B. Rudometova, D.N. Shcherbakov, A.P. Rudometov, A.A. Ilyichev, L.I. Karpenko

**Affiliations:** State Research Center of Virology and Biotechnology “Vector”, Koltsovo, Novosibirsk Region, Russia; State Research Center of Virology and Biotechnology “Vector”, Koltsovo, Novosibirsk Region, Russia; State Research Center of Virology and Biotechnology “Vector”, Koltsovo, Novosibirsk Region, Russia; State Research Center of Virology and Biotechnology “Vector”, Koltsovo, Novosibirsk Region, Russia; State Research Center of Virology and Biotechnology “Vector”, Koltsovo, Novosibirsk Region, Russia

**Keywords:** HIV-1, primary isolates, infectious molecular clones, env-pseudoviruses, virus neutralization assay, ВИЧ-1, первичные изоляты, инфекционные молекулярные клоны, env-псевдовирусы, анализ нейтрализации вируса

## Abstract

HIV infection still remains a major challenge for healthcare systems of the world. There are several aspects on counteracting the HIV/AIDS epidemic. The f irst aspect covers preventive measures including educational campaigns on HIV/AIDS and promotion of a healthy lifestyle, protected sex, and pre-exposure prophylaxis of vulnerable groups. The second aspect is timely HIV testing and the use of antiretroviral therapy when test results come back positive. The third aspect is the scientif ic research associated with discovering new pharmaceutical agents and developing HIV-1 vaccines. Selecting an adequate tool for quick and accurate in vitro eff icacy assessment is the key aspect for eff icacy assessment of vaccines and chemotherapy drugs. The classical method of virology, which makes it possible to evaluate the neutralizing activity of the sera of animals immunized with experimental vaccines and the eff icacy of chemotherapy agents is the method of neutralization using viral isolates and infectious molecular clones, i. e. infectious viral particles obtained via cell transfection with a plasmid vector including the full-length HIV-1 genome coding structural, regulatory, and accessory proteins of the virus required for the cultivation of replication-competent viral particles in cell culture. However, neutralization assessment using viral isolates and infectious molecular clones is demanding in terms of time, effort, and biosafety measures. An alternative eliminating these disadvantages and allowing for rapid screening is the use of pseudoviruses, which are recombinant viral particles, for the analysis of neutralizing activity. Pseudotyped viruses have defective genomes restricting their replication to a single cycle, which renders them harmless compared to infectious viruses. The present review focuses on describing viral model systems for in vitro eff icacy assessment of vaccines and drugs against HIV-1, which include primary HIV-1 isolates, laboratoryadapted strains, infectious molecular clones, and env-pseudoviruses. A brief comparison of the listed models is presented. The HIV-1 env-pseudoviruses approach is described in more detail

## Abstract (ru)

ВИЧ-инфекция по-прежнему остается одной из глобальных проблем здравоохранения во всем мире. Борьба с инфекцией ведется по нескольким направлениям. Во-первых, это профилактические мероприятия, которые включают просвещение населения по проблеме ВИЧ/СПИДа, пропаганду здорового образа жизни, защищенные половые контакты, доконтактную профилактику уязвимых групп населения. Во-вторых, прохождение своевременного тестирования на ВИЧ и применение антиретровирусной терапии в случае его обнаружения. В-третьих, это научные исследования, связанные как с поиском новых лекарственных агентов, так и с разработкой вакцины против ВИЧ-1. Ключевой момент при определении эффективности вакцин и химиотерапевтических препаратов – выбор инструмента, позволяющего быстро и точно оценить их эффективность in vitro. Классическим методом вирусологии, позволяющим оценить нейтрализующую активность сывороток животных, иммунизированных экспериментальными вакцинами, и эффективность химиотерапевтических агентов, является метод нейтрализации с использованием вирусных изолятов, а также инфекционных молекулярных клонов, которые представляют собой инфекционные вирусные частицы, полученные путем трансфекции клеток плазмидным вектором, содержащим полноразмерный геном ВИЧ-1, кодирующий структурные, регуляторные и вспомогательные белки вируса, необходимые для образования репликационно-компетентных вирусных частиц в культуре клеток. При этом метод нейтрализации с использованием вирусных изолятов и инфекционных молекулярных клонов отличается трудоемкостью, продолжительностью и требует повышенных мер биобезопасности. Альтернативным решением, устраняющим указанные недостатки и позволяющим проводить быстрый скрининг, является использование для анализа нейтрализующей активности псевдовирусов, которые представляют собой рекомбинантные вирусные частицы. В отличие от инфекционных вирусов, работа с псевдовирусами безопасна, поскольку геном псевдовирусов нарушен для того, чтобы их инфекция ограничивалась лишь одним циклом. Данный обзор посвящен описанию модельных вирусных систем, используемых для оценки эффективности вакцин и лекарственных препаратов против ВИЧ-1 in vitro: первичных изолятов ВИЧ-1 и лабораторно-адаптированных штаммов, инфекционных молекулярных клонов и env-псевдовирусов. Кратко представлена их сравнительная характеристика. Более подробно описана технология env-псевдовирусов ВИЧ-1.

## Introduction

The HIV/AIDS pandemic still remains a major problem for healthcare systems of the world with about two million newly infected individuals every year^1^. At present, antiretroviral therapy is the most common way to manage HIV infection, as it reduces viral loads and prolongs and improves the quality of life of HIV-infected patients. However, the currently available antiretroviral drugs also have major shortcomings, such as high costs, marked side effects, developing drug resistance, a necessity for regimen changes, and the life-long duration of the therapy (Arts, Hazuda, 2012). Above all that, we are yet to find the cure for HIV infection (Phanuphak, Gulick, 2020). As a result, the development of effective preventive vaccines against HIV/AIDS remains a top priority (Stephenson et al., 2020).

^1^ Fact Sheet on HIV/AIDS. World Health Organization, 2020. URL: https://www. who.int/ru/news-room/fact-sheets/detail/hiv-aids(Accessed June 2, 2021).

As of today, the RV144 clinical trials performed in Thailand from 2003 to 2009 are considered the most successful. The studied vaccine showed an efficacy of 60 % in 12 months after vaccination and 31.2 % – after a 3.5-year follow-up (Kim et al., 2015). Several years later the RV144 vaccine components were modified to express the antigens of the HIV strains circulating in South Africa. In January 2020, early results of clinical trials showed that the modified vaccine failed to prevent HIV-1 infection in volunteers (Gray et al., 2021). Nowadays, there are still numerous unresolved issues in HIV-1 vaccine development, yet it is clear that it is necessary to use new approaches to its design (Hsu, O’Connell, 2017), hence the intense research for the induction of the protective Т and В cell immune response to HIV-1, including broadly neutralizing antibodies (bnAbs) (Shcherbakov et al., 2015; Rudometov et al., 2019b; Jones et al., 2020; Liu et al., 2020; Ng’uni et al., 2020).

Selecting an adequate tool for in vitro efficacy assessment is an integral part of scientific research aimed at developing vaccines and chemotherapy drugs against viral pathogens, including HIV-1. The neutralizing activity of the sera from the animals immunized with experimental vaccines and the efficacy of chemotherapy agents are conventionally assessed using viral isolates (Jackson et al., 1988). However, this process is demanding in terms of time, effort, and biosafety measures. An alternative method is to use infectious molecular clones, i. e. infectious viral particles obtained via cell transfection with plasmid vector including the full-length HIV-1 genome coding structural, regulatory, and accessory proteins of the virus required for the cultivation of replication-competent viral particles in cell culture (Peden et al., 1991).

In recent years, many researchers give preference to the pseudotyped virus approach, a safer method suitable for BSL-2 lab settings (Li Q. et al., 2018; Montefiori et al., 2018). Compared to viral isolates and infectious molecular clones, pseudotyped viruses are harmless, because virus replication is restricted to a single cycle due to mutations in coding regions of the genome, which is why pseudotyped viruses are often called single-cycle viruses (Cheresiz et al., 2010; Li Q. et al., 2018).

HIV-1 model systems for in vitro efficacy assessment of chemotherapy drugs, bnAbs, and candidate vaccines against HIV-1 will be considered in the present review.

HIV-1 isolates and laboratory-adapted strains

Historically, HIV-1 primary isolates were the first system for analyzing vaccine efficacy and neutralizing the activity of antibodies (Jackson et al., 1988). Viral isolates are obtained via cocultivation of the peripheral blood mononuclear cells (PBMC) of an HIV-positive patient and the PHA-stimulated PBMC of a healthy donor. Here, the viruses isolated from blood appear as a genetically heterogeneous population due to the quasispecies nature of HIV-1. To eliminate possible selective pressure on viral isolates and ensure optimal preservation of a viral phenotype, the virus is cultivated using primary cell culture, rather than cell lines (Voronin et al., 2007; Van’t Wout et al., 2008). The presence of neutralizing antibodies in sera from vaccinated subjects or the efficacy of an antiviral agent is typically identified in a PBMC culture with an added infectious dose of the virus and serial dilutions of immune serum or tested compound. HIV-1 replication suppression is assessed using ELISA by measuring p24 content (structural component of HIV-1 capsid) in the culture medium (Zyryanova et al., 2020a).

However, the use of HIV-1 primary isolates for virus neutralization analysis has several shortcomings, including the use of primary PBMCs for pathogen replication, high biosafety requirements, low repeatability of the results, and therefore standardization issues (Mascola et al., 1996, 2005). Thus, some HIV-1 strains (IIIB/LAV, MN, SF2) were adapted for replication in immortalized cell lines (H9, CEM) for the sake of simplicity and to ensure repeatability of the experiments in the first years of vaccine development. These were later referred to as laboratory-adapted strains or, more accurately, T cell line adapted strains. Vaccination of volunteers with recombinant trimers based on laboratory-adapted HIV-1 strains induced the antibodies neutralizing these specific laboratory strains. The additional experiments involving HIV-1 primary isolates showed the absence of neutralizing activity against primary isolates, despite intense induction of neutralizing antibodies against the laboratory-adapted strains (Mascola et al., 1996; Montefiori et al., 2018). Apparently, the neutralization analysis performed using laboratory-adapted strains could produce misguiding results, and the researchers came back to primary isolates as a more adequate tool for analyzing the virus-neutralizing activity of the antibodies induced as a result of vaccination. Since the method is labor-intensive and does not allow for mass analysis, it began to be used for the concluding stages of research.

HIV-1 infectious molecular clones

**Fig. 1.**
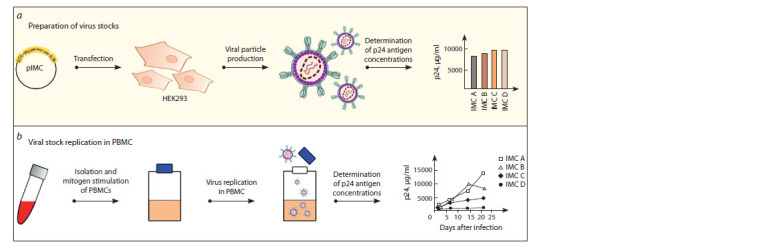
HIV-1 IMC technology

Taking into account the cultivation difficulties and significant heterogeneity of HIV-1 primary isolates and laboratory-adapted strains, as well as the variability of donor PBMCs (Polonis et al., 2008), HIV-1 infectious molecular clones (IMCs) were chosen for consistent replication of viral particles. IMCs are obtained via cell transfection with a plasmid vector including a full-length HIV-1 genome to ensure the generation of replication-competent viral particles in a eukaryotic cell culture (Fig. 1). Compared to HIV-1 primary isolates, this approach makes it possible to obtain genetically homogeneous viral particles, since an HIV-1 genome is present in the plasmid vector in the form of DNA (Edmonds et al., 2010; Zyryanova et al., 2020b). To ensure standardization of neutralization analysis using IMCs, modified continuous cell lines with a cell-surface CD4 receptor and CCR5 and CXCR4 co-receptors were genetically engineered (Princen et al., 2004; González et al., 2009). Since IMCs are essentially infectious viral particles, the relevant biosafety requirements are to be fulfilled, similarly to primary isolates and laboratory-adapted strains, and the analysis itself is rather time-consuming.

Conditionally, HIV-1 IMCs are obtained in two stages. At the f irst stage (a), viral particles, also referred to as virus stock, are produced via HEK293 cell line transfection. At the second stage (b), the virus stock is further replicated for several weeks using PHA-stimulated PBMCs from a healthy donor. The titers of viral particles are measured at each stage using the ELISA based on р24 antigen content in the culture medium. A replication-competent virus is produced if p24 capsid protein content becomes at least 1000 times as high as its initial content in the culture medium.

At the same time, the use of IMCs makes it possible to characterize and study biological properties of genetically different HIV-1 isolates (Ochsenbauer et al., 2012; Baalwa et al., 2013; Wang et al., 2013; Chenine et al., 2018; Zyryanova et al., 2020b), investigate the development mechanisms of drug-resistant HIV-1 strains and the effect of mutations on the biological properties of the virus (Johnston et al., 2005; Pugach et al., 2007; Varghese et al., 2013), and discover new antiretroviral agents (Su et al., 2019; Wagstaff et al., 2019; Mavian et al., 2020).

HIV-1 env-pseudoviruses

The use of classical virological methods to work with HIV-1 faces a number of difficulties noted above. Env-pseudovirus technology has proved to be a potent tool for quick and adequate assessment of humoral immune response to vaccine constructs and screening of potential chemotherapeutic agents, specifically entry inhibitors (Montefiori et al., 2018).

HIV-1 env-pseudoviruses are recombinant viral particles obtained via eukaryotic cell transfection with the two plasmids referred to as core and envelope. The core plasmid includes genes of structural (Gag and Pol), regulatory (Tat and Rev), and accessory (Vpu, Vpr, Vif, and Nef) HIV-1 proteins necessary for viral particle assembly, as well as sequences required for viral RNA packaging (Ψ). The envelope plasmid carries an envelope glycoprotein gene (Env) of certain HIV-1 subtype. As a result of transfection, viral particles with a defective genome incapable of assembling infectious daughter virions are obtained (Li M. et al., 2005; Li Q. et al., 2018). Electron microscopy studies show that the HEK293 cell line transfection with two plasmids produces viral particles morphologically identical to the HIV-1 virions (Zaitsev et al., 2019; Ladinsky et al., 2020).

**Fig. 2.**
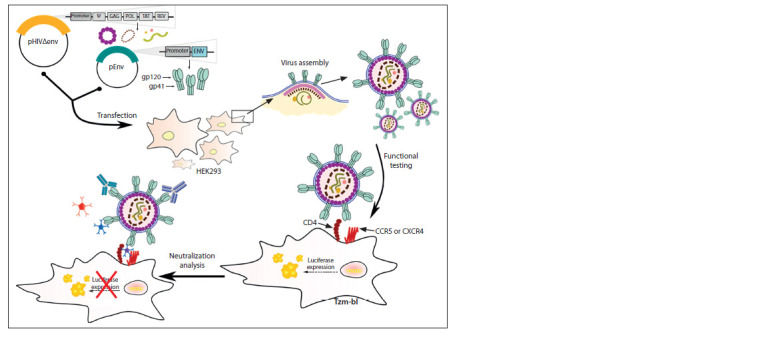
HIV-1 env-pseudovirus technology.

The determination of the functional activity of env-pseudoviruses and analyses neutralization are carried out on a TZM-bl cell line, which is a continuous, genetically modified HeLa cell line with cell-surface CD4 receptors and CCR5 and CXCR4 co-receptors. In addition, firefly luciferase and β-galactosidase E. coli reporter genes are integrated into the TZM-bl cell line genome under transcriptional control of HIV-1 long terminal repeat. When a pseudotyped virus enters the target TZM-bl cell, synthesis of a viral Tat protein triggers luciferase reporter gene expression detectable by a luminometer. Here, high luminescence intensity indicates that pseudotyped viral particles have entered target cells, whereas suppressed luminescence indicates that the HIV-1 env-pseudoviruses have been neutralized (Platt et al., 1998; Wei et al., 2002). A general work technique of env-pseudovirus system is shown in Fig. 2.

Experimental study of env-pseudoviruses is done in several stages: Stage 1 includes viral particle assembly via HEK293 cell line transfection with two plasmids, referred to as core and envelope; at Stage 2, one measures the functional activity of pseudotyped viral particles, i. e. their ability to infect target cells and trigger firefly luciferase reporter gene expression; at Stage 3 the neutralization level is analyzed using immune sera or chemotherapeutic agents to measure their ability to block pseudotyped virus entry to target cells.

An env-pseudovirus system has a number of distinct advantages. First, since TZM-bl is a stable continuous cell line, it may be used as a substitute for human primary Т cells, thereby reducing the need for individual donor cells. Second, env-pseudoviruses are harmless compared to viral isolates and IMCs requiring higher biosafety levels, which makes experimental studies more complicated and expensive. Third, Env protein forms trimer structures at the surface of pseudotyped viral particles, which are identical to those of the natural virus. However, the main advantage of the pseudotyped virus technology is that it makes it possible to obtain the equivalents of the viral particles of various HIV-1 subtypes and strains, thereby providing broad coverage of HIV-1 genetic diversity (Seaman et al., 2010; Montefiori et al., 2018). In addition, the neutralization assessment method using env-pseudoviruses favors further optimization and standardization (Wei et al., 2002; Seaman et al., 2010; Sarzotti-Kelsoe et al., 2014). A brief comparison of HIV-1 primary isolates and laboratory- adapted strains, IMCs, and env-pseudoviruses is presented in the Table.

**Table 1.**
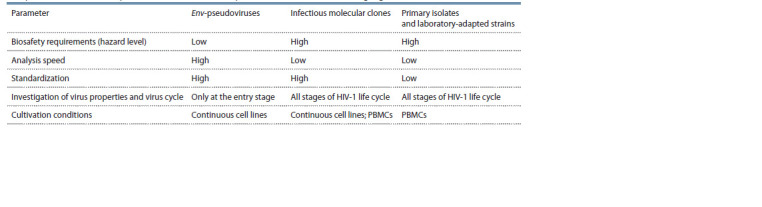
Comparison of HIV-1 model systems used for in vitro efficacy assessment of vaccines and drugs against HIV-1

It should be noted that the protocols and recommendations for neutralization assessment using env-pseudoviruses are available at the website of the Los Alamos National Laboratory https://www.hiv.lanl.gov/content/nab-reference-strains/html/home.htm/ (). In addition, the HIV Reagent Program supported by the National Institute of Allergy and Infectious Diseases and curated by the National Collection of Type Cultures makes it possible to obtain all the components (cell lines, plasmids, monoclonal antibodies) required for implementing the technology.

Here are several noteworthy applications of env-pseudoviruses panels. Antiviral activity of clinically approved co-receptor antagonist Maraviroc was demonstrated using 160 HIV-1 subtype B env-pseudoviruses and 40 env-pseudoviruses of other HIV-1 subtypes (Dorr et al., 2005). The activity of Ibalizumab, a monoclonal antibody binding to the CD4 receptor, was demonstrated using 116 env-pseudoviruses of subtypes A, B, C, and CRF01_AE (Pace et al., 2013). HIV-1 env-pseudoviruses panels were also used to investigate the bnAbs spectrum with respect to various genetic variants of HIV-1. For instance, the neutralization breadth of 98 % for bnAb 10E8 was demonstrated using a panel of 181 env-pseudoviruses of subtypes A, B, C, D, G, CRF01_AE, and CRF02_AG (Huang et al., 2012); neutralization breadth of 91 % for bnAb VRC01 was demonstrated using 196 env-pseudoviruses (Wu X. et al., 2010); neutralization breadth of 49 % for bnAb VRC34.01 was demonstrated using 179 env-pseudoviruses (Kong et al., 2016). It is the introduction of pseudotyped virus panels, including a wide range of genetically diverse HIV-1 variants, that led to a breakthrough in the production and characterization of monoclonal broadly neutralizing antibodies.

Env-pseudoviruses panels are extensively used to study the humoral immune response induced by candidate vaccines against HIV-1 at a design stage and during pre-clinical and clinical trials, since the presence of virus-neutralizing antibodies in the vaccinated subjects is among the key indicators of HIV vaccine effectiveness (Rudometov et al., 2019a; Ou et al., 2020). Recent papers by Xu et al., who developed a vaccination regimen based on fusion peptide (FP) of gp41, a key structural component of HIV-1, may be cited as an example. Earlier, they identified the VRC34.01 antibody from an HIV-positive donor, which was aimed at the conservative N-terminal region of HIV-1 FP. Since FP is a short linear peptide, it has low natural immunogenicity, which is why garden snail hemocyanin widely used in biotechnology was used as a carrier protein. Immunization of laboratory animals by an FP bound to garden snail hemocyanin with subsequent boosting by a BG505 trimer resulted in induction of antibodies with neutralization breadth of 31 % demonstrated using a panel of 208 env-pseudoviruses of various HIV-1 subtypes (Xu et al., 2018).

In conclusion of this review, it should be mentioned that an HIV-1 pseudotyping system is tolerant to incorporation of surface proteins of various enveloped viruses. Since most laboratory experiments and studies involving viruses are to be performed in BSL-3 or BSL-4 lab settings, the use of pseudotyped viruses instead of wild-type ones makes it possible for various research groups to study viruses of interest and design antiviral drugs and vaccines against highly dangerous viruses. For example, HIV-1 pseudotyping system was used to obtain the viral particles carrying surface glycoproteins of Ebola virus (Mohan et al., 2015), Marburg virus (Zhang L. et al., 2019), Lassa fever (Zhang X. et al., 2019), Middle East respiratory syndrome coronavirus (Zhao et al., 2013), Rabies virus (Nie et al., 2017), Chikungunya virus (Wu J. et al., 2017), and Nipah virus (Nie et al., 2019). In addition, this technology is extensively used in designing pseudotyped virus platforms for SARS-CoV-2 (Hu et al., 2020; Hyseni et al., 2020; Johnson et al., 2020).

## Conclusion

All technologies considered above have their own advantages and shortcomings and most certainly complement each other in integrated studies. Despite the labor-intensity of primary isolate and IMC technologies in neutralization assessments, these models still remain valuable tools for investigating the biological properties of viruses. However, env-pseudovirus technology has currently become the base method for efficacy assessment of HIV-1 vaccines and antiviral agents (potential entry inhibitors). Its main advantages include safety, high repeatability of the results, standardization potential, and ability to work with virus particles exposing surface glycoproteins of multiple virus subtypes.

## Conflict of interest

The authors declare no conflict of interest.
